# ASSOCIATION OF HEARING LOSS AND INCIDENT PARKINSON’S DISEASE: A COHORT STUDY IN US VETERANS

**DOI:** 10.1093/geroni/igae098.2824

**Published:** 2024-12-31

**Authors:** Lee Neilson, Kelly Reavis, Jack Wiedrick, Gregory Scott

**Affiliations:** Oregon Health & Science University, Portland, Oregon, United States; VA Portland Healthcare System, Portland, Oregon, United States; Oregon Health & Science University, Portland, Oregon, United States; Portland VA Medical Center, Portland, Oregon, United States

## Abstract

Parkinson’s disease (PD) is a heterogeneous disease with non-motor manifestations that often precede motor symptom onset. Sensory disturbances such as loss of sense of smell and visual problems are well-characterized, but the impact of hearing loss on PD incidence remains unclear. We undertook an electronic health record-based cohort study of US military Veterans with objective audiogram data (n=3,596,365), using inverse probability of treatment weighting to calculate cumulative incidence of PD, adjusted for competing risk of death and key confounders. Risk difference and 95% confidence intervals (CI) were estimated by hearing loss severity. We also analyzed how this varied when combined with known comorbid conditions and receiving a hearing aid. Results suggest that hearing loss increases the risk of developing PD in a dose-dependent manner; worse hearing confers a higher risk. The number of additional cases of PD per 10,000 person-years at 5-years due to mild, moderate, moderately-severe, and severe-to-profound hearing loss was 2.0 (95% CI: 0.9, 3.1), 5.4 (95% CI: 3.7, 7.1), 5.8 (95% CI: 3.3, 8.3), and 10.6 (95% CI: 4, 17.2), respectively. Hearing loss also synergized with known prodromal disorders, increasing PD risk more than additively. Dispensation of a hearing aid within two years of the audiogram attenuated the risk of incident PD; the reduction in cases of PD per 10,000 person-years at 5-years was -9.2 (95% CI: -10.7, -7.7). Hearing loss appears to be an independent risk factor for development of PD. Widespread screening for hearing loss and appropriate use of hearing aids may attenuate this risk.

